# Managing Student Well‐Being After Social Upheaval: An Examination of Bangladeshi University Responses

**DOI:** 10.1002/puh2.70187

**Published:** 2026-01-22

**Authors:** Taha Husain

**Affiliations:** ^1^ Department of Gender and Development Studies Begum Rokeya University Rangpur Bangladesh

**Keywords:** coping mechanisms, mental health, reconciliation, students’ movement, university support systems, youth activism

## Abstract

**Purpose:**

This study investigates the psychological and academic impacts of the July Students’ Movement on Bangladeshi university students. It aims to understand their mental health, academic performance, coping strategies, and perceptions of university‐led mental health and reconciliation programs.

**Methodology:**

A cross‐sectional survey design was used, gathering data from 464 Bangladeshi university students from both public and private institutions. A convenience sampling approach was employed. Descriptive statistics summarized key variables, and correlation and regression analyses were conducted to identify relationships and significant predictors of psychological responses.

**Findings:**

Results showed that 81.9% of participants were movement activists, reporting high anxiety (*M* = 4.2), sleep disturbances (*M* = 3.6), and isolation (*M* = 3.5). Although students mainly relied on friends and family for support (*M* = 4.1), engagement with formal counseling was limited (*M* = 2.5), indicating barriers related to access or stigma. University efforts to rebuild relationships (*M* = 3.9) and promote unity (*M* = 4.0) were appreciated, along with strong support for improved mental health programs (*M* = 4.2). Older students experienced less anxiety from social media rumors (*r* = −0.22) and safety concerns (*r* = −0.20), whereas female students reported higher stress levels (*r* = 0.18 for rumors). Gender (*p* = 0.028) and study level (*p* = 0.022) emerged as significant predictors of psychological responses.

**Conclusion:**

This study provides unique insights into the specific psychological and academic effects of student activism in Bangladesh after the July Movement. It highlights the disparity between informal and formal support‐seeking behaviors and provides empirical data on perceptions of university reconciliation efforts.

## Graphical abstract

Bangladesh's 2024 student movement triggered widespread anxiety, sleep disturbances, and isolation among university activists, who relied heavily on peers rather than formal counseling. Findings highlight the urgent need for culturally sensitive, student‐centered mental health and reconciliation programs.

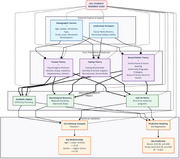

## 1 Introduction

Youth‐led movements have long been powerful catalysts for social and political transformation, with young activists driving demands for justice, rights, and institutional reform [1]. From the pivotal role of youth in the civil rights movements of the 1960s [2] to contemporary global climate strikes [3], these movements underscore the agency and resilience of young people in confronting systemic inequities [4]. However, activism entails significant psychological consequences, presenting a complex duality: Although it can foster empowerment and collective efficacy [5], it also risks causing stress, anxiety, and trauma [6].

The July Students’ Movement of 2024 in Bangladesh offers a critical case study for examining these psychological effects, particularly given the limited research on university students’ experiences in sociopolitical movements in the Global South. Young activists, still navigating identity formation and emotional regulation, face heightened vulnerability to psychological stressors, making their mental health outcomes a vital area of study. Thus, this study aims to (1) assess the psychological impacts (e.g., anxiety and sleep disturbances) of the July Students’ Movement on Bangladeshi university students; (2) examine their coping mechanisms and reliance on informal versus formal support; (3) evaluate perceptions of university‐led reconciliation and mental health initiatives; and (4) explore demographic influences on psychological responses.

## 2 Literature Review

Youth activism has garnered significant attention in recent scholarship due to its profound psychological and social implications. This review synthesizes research on the psychological impact of youth activism, factors influencing mental health outcomes, coping mechanisms, support systems, and implications for policy and practice. The literature highlights the dual nature of activism: It is both empowering and taxing, with significant variations depending on context, identity, and systemic factors.

### 2.1 Psychological Impact of Youth Activism

The psychological consequences of youth activism reveal a fundamental duality consistently documented across diverse contexts and populations. Although activism can foster empowerment, agency, and identity development among young people, it also carries substantial risks to mental and physical well‐being [7, 8]. This duality is particularly evident in the development of critical consciousness. Although awareness of systemic oppression can be liberating and empowering, confronting structural inequities may trigger stress, anxiety, and other adverse mental health outcomes [9, 10].

Research with youth activists suggests that although participants generally report more benefits than costs from their activism, the negative impacts have serious consequences for their overall well‐being [11]. The costs of activism are significantly associated with worse mental health, physical health, and reduced flourishing, whereas benefits primarily correlate with increased flourishing [11]. Studies find that movements involving repression, confrontation, or perceived failure particularly amplify activists’ psychological distress [12]. Activist burnout has emerged as a significant threat not only to individual well‐being but also to the sustainability and effectiveness of social movements [12, 13, 14].

Young activists face three primary sources of burnout: backlash in response to their efforts, pressure to be the “savior generation,” and the slow pace of change [11]. The relationship between civic engagement and mental health is nonlinear, with the emotional costs of activism—including additional stressors and responsibilities—potentially leading to psychological distress, especially when addressing challenging social issues [15, 16]. Marginalized youth activists face additional challenges, as their experiences with discrimination and systemic oppression can compound the typical stressors of activism. For example, research on Black and Latinx emerging adults shows that racism‐based events can create traumatic stress with long‐term mental health consequences, necessitating specific approaches to healing and wellness [17]. Without effective interventions to address emotional well‐being, the risks of disengagement and burnout increase significantly among young activists [16].

### 2.2 Factors Influencing Mental Health Outcomes

The type and context of civic engagement activities significantly influence the mental health outcomes of youth activism. Research shows that different forms of activism produce varying psychological effects. Although volunteering and voting are positively associated with subsequent mental health and positive health behaviors, activism is linked explicitly to more health‐risk behaviors and shows no clear association with improved mental health [18]. However, engagement in positive activities such as advocacy efforts, volunteering, and altruism following traumatic events can foster post‐traumatic growth and positively impact mental well‐being, as demonstrated in studies of community responses to disasters and pandemic mutual aid efforts [19, 20, 21].

Individual characteristics, particularly marginalized identities, create significant variations in activism's mental health impact. Research reveals that the costs of activism correlate directly with the number of marginalized identities youth activists hold, with queer Black girls reporting the highest overall costs and queer multiracial girls showing the highest rates of burnout [22]. Students facing legal vulnerability, such as undocumented students, experience hefty tolls on their mental health from activism [23, 24]. The developmental context plays a crucial role in determining outcomes. Positive Developmental Theory suggests that participation in supportive developmental contexts, such as communities and schools, can foster healthy youth development through empowerment, greater self‐esteem, social support, and fewer depressive symptoms [19, 20].

Climate activism research indicates that although young people experience unique combinations of disempowerment and climate distress that can negatively impact their well‐being, engaging in climate action with proper support can foster self‐ and collective resilience, efficacy, and agency [25, 26]. Organizational and systemic factors significantly impact mental health outcomes. Organizational structures and features can either facilitate activism and create supportive environments for young people from marginalized communities or become sources of additional costs and burnout [22, 27].

University mental health programs, crisis response protocols, flexible academic policies, and strengthened social support networks are essential for supporting student well‐being, particularly given that resilience serves as a crucial protective factor in coping with challenges [28, 29]. Policy interventions addressing the root causes of oppression can significantly impact the mental health outcomes of activists. For immigrant‐origin student activists, policies that curtail local immigration enforcement and create pathways to citizenship would likely reduce anxiety and improve mental well‐being by addressing the structural inequities that fuel distress [23, 30].

### 2.3 Coping Mechanisms and Support Systems

Research indicates that a sense of belonging to an activist community serves as a significant protective factor for mental health, physical well‐being, and overall flourishing among young activists [11]. This community connection helps buffer against the three primary sources of burnout: backlash in response to their efforts, pressure to be the “savior generation,” and the slow pace of change [11]. Resilience emerges as a crucial protective factor in helping university students cope with challenges and adversity [28, 29]. Building resilience can empower students to navigate uncertainty, setbacks, and transitions more effectively, fostering adaptive coping strategies and strengthening social support networks [28, 29].

For young activists from marginalized communities, tailored mental health resources, including counseling services and therapy, should be easily accessible [7, 8]. Collective care practices, such as peer support groups and community‐building activities, offer important avenues for activists to connect, share experiences, and address burnout together [7, 17]. Research indicates that Black and Latinx emerging adults utilize cultural, ancestral, spiritual, and religious practices as healing approaches, underscoring the significance of radical hope and radical healing for these communities [17]. Effective program design requires integrating strategies that support youth mental health alongside civic engagement activities.

Without interventions to address emotional well‐being, such as relaxation and self‐care approaches, the risks of disengagement and burnout increase significantly [15, 16]. Programs should offer mentorship, self‐care workshops, and access to counseling services to prevent burnout and ensure that youth can sustain their activism without adverse consequences [16]. At the institutional level, universities should implement comprehensive mental health programs, develop crisis response protocols, promote mental health awareness and education, strengthen social support networks, and adopt flexible academic policies [28]. Prioritizing mental health within activist communities not only supports individual well‐being but also enhances the sustainability of activism efforts [7].

### 2.4 Implications for Policy and Practice

The evidence highlights several critical areas where practice and policy interventions can better support the mental health and sustainability of youth activists, for legally vulnerable populations, such as undocumented students, wellness strategies must include equitable allocation of emotional, financial, and legal support, whereas long‐term solutions require addressing the roots of oppression through policies that curtail local immigration enforcement and create pathways to citizenship [23, 24, 30]. Mental health interventions must be tailored to the specific needs of marginalized activists, with easily accessible counseling services and therapy, alongside collective care practices such as peer support groups and community‐building activities that allow activists to connect, share experiences, and collectively address burnout [7, 17].

Research indicates that Black and Latinx communities, in particular, benefit from cultural, ancestral, spiritual, and religious healing practices that emphasize radical hope and radical healing [17]. Organizations must recognize that their structures and features can either facilitate activism and create supportive environments or become sources of additional costs and burnout, particularly for young people from marginalized communities [22, 27, 31, 32, 33]. Given that the costs of activism correlate with the number of marginalized identities activists hold, organizations should provide multiple avenues for engagement to sustain involvement while balancing the costs of forms of activism that disproportionately contribute to burnout [22, 27].

At the institutional level, universities and policymakers should implement comprehensive mental health programs, develop crisis response protocols, promote mental health awareness and education, strengthen social support networks, and adopt flexible academic policies [28, 29, 34]. Building resilience serves as a crucial protective factor that empowers students to navigate uncertainty, setbacks, and transitions more effectively while fostering adaptive coping strategies [28, 29]. Program design must integrate strategies that support youth mental health alongside civic engagement activities, including mentorship, self‐care workshops, and access to counseling services to prevent burnout and ensure sustainable activism [15, 16]. Research shows that without effective interventions to address emotional well‐being, the risks of disengagement and burnout increase significantly [15, 16].

Finally, recognizing the power of youth activism to catalyze policy shifts toward equity and sustainability, policymakers should create inclusive frameworks that acknowledge the needs of indigenous and vulnerable populations and support participatory, equity‐focused governance approaches [35, 36, 37]. These comprehensive approaches can maximize the benefits of activism while mitigating risks, ultimately sustaining activists’ efforts for social transformation. This study addresses these gaps by examining the July Students’ Movement of 2024, offering empirical insights into the mental health challenges and recovery processes of student activists. By integrating findings on the psychological impact of activism, coping strategies, and reconciliation, this research contributes to broader discussions on mental health, youth political engagement, and the sustainability of activist movements. These findings have implications for policymakers, educators, and mental health professionals seeking to develop policy interventions that support the well‐being and long‐term engagement of young activists in sociopolitical change.

## 3 Theories and Research Framework

This study integrates trauma theory, coping theory, and reconciliation theory to investigate the psychological effects of the July Student Movement on university students. These theories provide a comprehensive lens for examining the psychological, emotional, and social aspects of students’ experiences during and after the movement. Below, each theory is discussed in detail, followed by a table mapping survey questions developed from these constructs.

### 3.1 Trauma Theory

Trauma theory examines the psychological and emotional consequences of exposure to traumatic events [38]. It provides a framework for understanding mental health disorders like PTSD, anxiety, and depression. Contemporary research has expanded its applications, particularly in educational settings, with a focus on trauma‐informed care (TIC). Recent scholarship, including Lembke et al. [39], has developed taxonomies of trauma‐sensitive practices and reviewed the impact of trauma‐informed approaches on academic achievement. Modern trauma theory integrates various frameworks and emphasizes social‐ecological perspectives, moving beyond individual pathology to include broader educational and social contexts [40, 41].

### 3.2 Coping Theory

Lazarus and Folkman's [42] coping theory examines how individuals manage stress and adversity, differentiating between adaptive and maladaptive coping mechanisms. Contemporary research has addressed theoretical inconsistencies and measurement issues by recognizing the reciprocal relationship between stress and coping, as well as changes in social environments [43, 44]. Recent studies have highlighted the detrimental effects of stress on college students, associating it with mental health issues [45, 46]. Modern coping theory integrates multiple frameworks, emphasizing the importance of belongingness and community connections to enhance coping self‐efficacy [47]. It now incorporates social‐ecological perspectives and examines community‐level interventions [40, 41, 48].

### 3.3 Reconciliation Theory

Reconciliation theory, rooted in conflict resolution, is crucial for understanding post‐conflict healing processes. Although useful, it has been critiqued for overlooking individual psychological needs [49]. Contemporary scholarship combines trauma, coping, and reconciliation frameworks to address mental health challenges. Chen et al. [50] developed a chain‐mediation model to examine the roles of school climate, belonging, and social avoidance patterns in the development of negative emotions.

### 3.4 Research Framework

**FIGURE 1:**
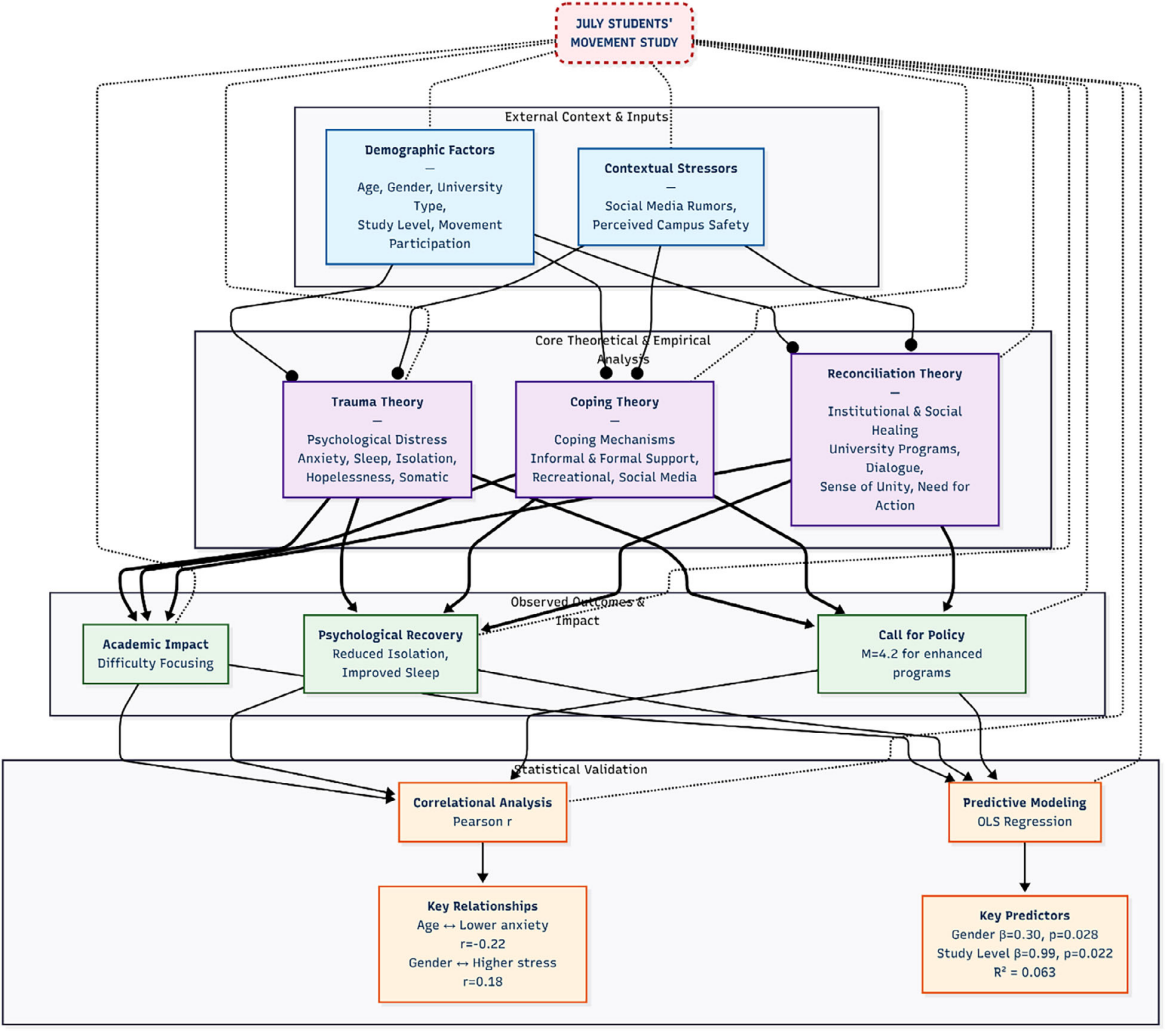
Theoretical and empirical research framework of the July Students’ Movement Study. *Source:* Author's creation.

Modern research has integrated multiple theoretical models to understand complex student mental health issues. Yao et al. [51] and Hou et al. [52] examined the interactions among perceived social support, psychological resilience, and coping styles among university students. Jameel et al. [53] examined the role of parent–child relationships and social support systems in maintaining mental well‐being after the pandemic. Studies have demonstrated a bidirectional relationship between mental health and academic performance, underscoring the importance of maintaining optimal mental health for academic success [54, 55]. Muldoon et al. [56] incorporated social identity theory to understand coping and recovery outcomes. Figure 1 presents an integrated flowchart of the research framework, mapping demographic antecedents and contextual stressors through trauma, coping, and reconciliation theories to psychological and academic outcomes. Survey items are embedded within each theoretical domain, with significant correlation and regression pathways indicated.

## 4 Methodology

This study employed a systematic, ethical, and rigorous approach to investigate the psychological and academic impacts of the July Students’ Movement on university students in Bangladesh. A quantitative survey design was used to collect and analyze data, ensuring objective measurement of students’ emotional well‐being, coping strategies, and perceptions of institutional support. Ethical considerations were prioritized to protect participants’ rights and well‐being, enhancing the study's integrity.

### 4.1 Research Design

A cross‐sectional survey design was used to capture students’ immediate psychological and social responses following the July Students’ Movement. This approach enabled the objective measurement and statistical analysis of variables, including anxiety, coping mechanisms, and perceptions of university support, facilitating the identification of patterns across a diverse student population. The design aligns with trauma, coping, and reconciliation theories by quantifying psychological distress, adaptive strategies, and institutional support perceptions, respectively. Structured questionnaires ensured consistent data collection, enhancing the reliability and validity of the findings.

### 4.2 Sampling Strategy

The study targeted currently enrolled university students aged 18–30 from public and private institutions in Bangladesh, encompassing diverse demographics (age, gender, and academic level). Convenience sampling was employed to recruit 464 participants through university email lists, student associations, and social media announcements, striking a balance between feasibility and representativeness in the post‐movement recovery context. This method ensured access to students directly or indirectly affected by the movement, including both participants and nonparticipants. Although convenience sampling limits the broad generalizability of the results, the sample size and diversity provided robust insights into students’ experiences.

### 4.3 Data Collection Tools and Procedures

A structured questionnaire, administered via Google Forms, was the primary data collection tool. The survey was developed through a multistep process to ensure theoretical alignment and contextual relevance. First, we reviewed validated scales from existing youth activism and mental health literature (e.g., [11, 38, 42, 49]), including items from established instruments like the Generalized Anxiety Disorder scale (for trauma‐related distress), the Brief COPE inventory (for coping mechanisms), and reconciliation‐focused surveys from post‐conflict studies (e.g., [49]). These were adapted for the Bangladeshi sociopolitical context, incorporating local cultural nuances such as the stigma around formal mental health services and the role of social media in movements. Questions were mapped directly to the three core theoretical frameworks: Trauma theory guided items on psychological distress and trauma responses; coping theory informed items on adaptive/maladaptive strategies and support‐seeking; and reconciliation theory shaped items on institutional support, unity, and healing processes. This mapping ensured comprehensive coverage of the study's aims, with Likert‐scale items (1 = strongly disagree to 5 = strongly agree) used for quantifiable responses.

To visualize this alignment, Table 1 buckets the macro frameworks into large categories (e.g., psychological distress under trauma theory), subcategories (e.g., emotional responses and physical manifestations), and representative survey items/questions. Not all survey items are listed here for brevity; the full instrument included 25 core items across these buckets, plus demographics.

**TABLE 1:** Mapping of theoretical frameworks to survey categories and items.

Macro framework	Large bucket (category)	Sub‐bucket	Representative survey items/Questions (examples)
**Trauma Theory** (focus: psychological and emotional consequences of traumatic events; e.g., [38])	Psychological impacts	Emotional responses	‐ I experience high anxiety or stress when recalling events from the July Students’ Movement. (*M* = 4.2) ‐ I feel a sense of hopelessness about the future after the movement. (*M* = 3.7) ‐ I experience anxiety from social media rumors related to the movement. (*M* = 4.1)
		Physical/Somatic manifestations	‐ I have experienced sleep disturbances since the movement. (*M* = 3.6) ‐ I have had headaches or other physical symptoms due to stress from the movement. (*M* = 3.4)
		Social/Environmental perceptions	‐ I feel isolated or lonely after the movement. (*M* = 3.5) ‐ I perceive the campus as unsafe following the movement. (*M* = 3.9)
**Coping Theory** (focus: management of stress through adaptive/maladaptive mechanisms; e.g., [42])	Coping mechanisms	Informal support‐seeking	‐ I have sought support from friends or family to cope with the movement's effects. (*M* = 4.1)
		Adaptive strategies	‐ I have used coping strategies like meditation, exercise, or hobbies. (*M* = 3.8) ‐ I have engaged in recreational activities to manage stress. (*M *= 3.7)
		Maladaptive/Formal strategies	‐ I have spent excessive time on social media as a way to cope. (*M* = 4.0) ‐ I have participated in formal counseling or therapy sessions. (M = 2.5)
**Reconciliation Theory** (focus: post‐conflict healing and institutional support; e.g., [49])	University initiatives and reconciliation	Institutional programs	‐ The university's reconciliation programs have helped rebuild relationships. (*M* = 3.9) ‐ The university has provided adequate support programs for affected students. (*M* = 3.7)
		Community and dialogue	‐ Participating in university dialogues has improved my mental health. (*M* = 3.5) ‐ I feel a sense of unity with my peers after the movement. (*M* = 4.0)
		Future needs	‐ There is a need for more university initiatives on mental health and reconciliation. (*M* = 4.2) ‐ Student perspectives should be included in reconciliation efforts. (*M* = 3.8)

*Note:* Items were measured on a 5‐point Likert scale (1 = strongly disagree, 5 = strongly agree). Means (*M*) are from Table 2 for reference. This mapping ensures the survey directly operationalizes the frameworks, allowing for targeted analysis of trauma responses, coping efficacy, and reconciliation outcomes.

*Source:* Author's creation.

**FIGURE 2:**
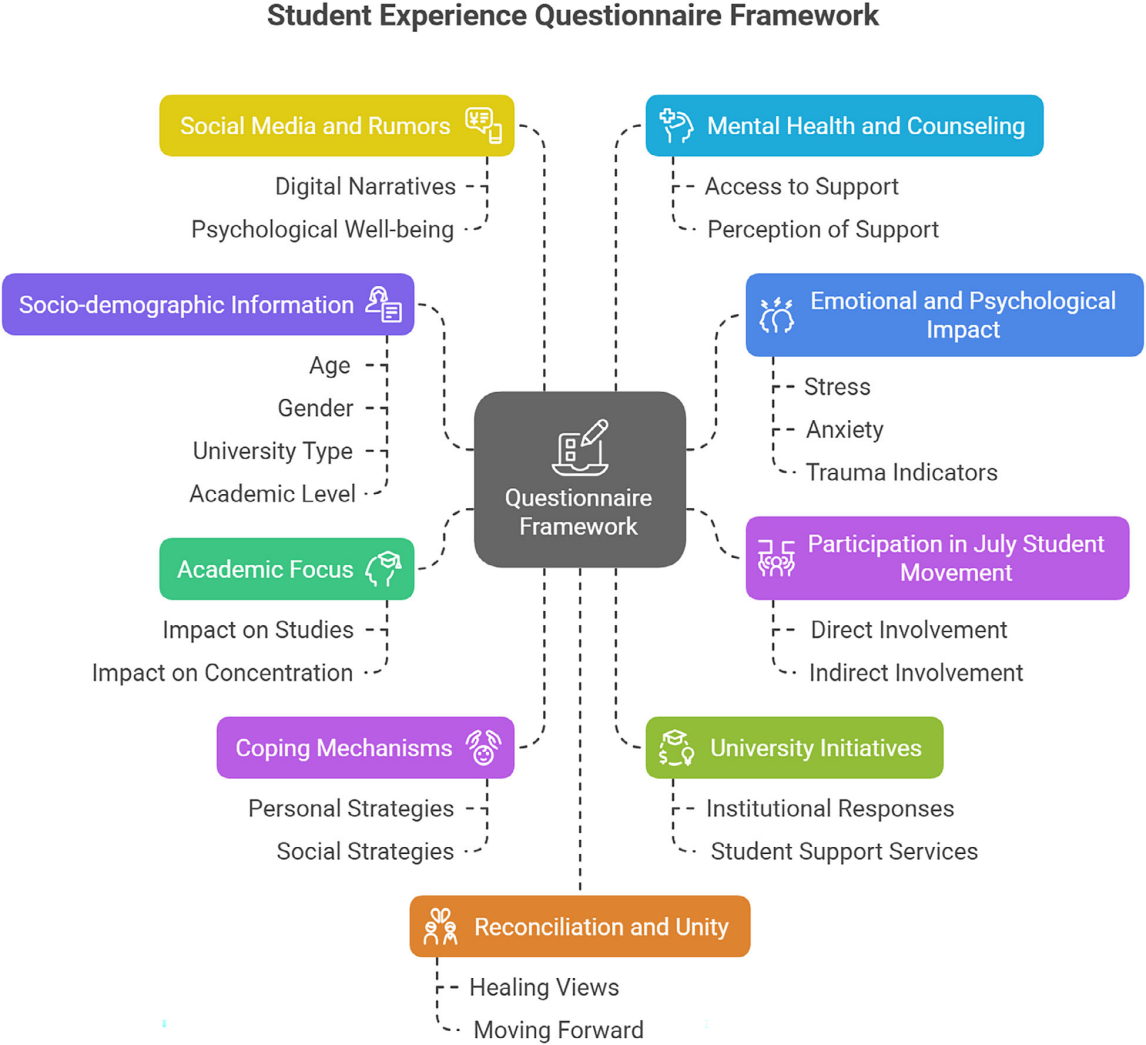
Questionnaire framework. *Source:* Author's creation.

The questionnaire was organized into sections (see Figure 2) and pretested with 20 students to ensure clarity and cultural relevance, with minor revisions made based on feedback (e.g., simplifying language around social media use to reduce ambiguity).

Data were collected over 3 weeks, with reminders sent to maximize response rates without coercion. Participants received clear instructions and an informed consent form detailing the study's purpose, procedures, and their right to withdraw. The online format ensured accessibility, particularly in the post‐movement context, enabling students across diverse institutions to participate.

### 4.4 Data Analysis

Quantitative data were analyzed using descriptive and inferential statistical methods. Descriptive statistics (frequencies, percentages, and *M* scores) summarized sociodemographic characteristics, emotional responses, and coping strategies. Inferential analyses, including Pearson correlation and OLS regression, examined relationships between demographic factors (e.g., age and gender) and psychological outcomes, with assumptions of normality and linearity verified. Missing data (<5%) were handled using listwise deletion, and outliers were assessed via boxplot analysis in SPSS. Analyses were conducted using SPSS for statistical computations, Python for data visualization (e.g., heatmaps and bar charts), and Microsoft Excel for preliminary organization, ensuring precision and reliability.

### 4.5 Ethical Considerations

Ethical integrity was a central aspect of the study. Participants provided informed consent, with a clear explanation of the study's objectives, its voluntary nature, and the confidentiality measures in place. No personally identifiable information was collected, and responses were stored securely on password‐protected servers. The questionnaire avoided sensitive or triggering questions to minimize psychological distress, and participants were provided with contact information for mental health resources. The research team adhered to principles of transparency, acknowledging potential limitations, such as sampling bias, and ensured responsible reporting of findings. Ethical approval was obtained from the university's institutional review board, reinforcing the study's commitment to participant well‐being and academic standards.

## 5 Results and Findings

This study examines the psychological and academic impacts of the July Students’ Movement, as well as coping strategies, perceptions of university support, and demographic influences. The findings reveal significant emotional distress, heavy reliance on informal support, moderate approval of institutional initiatives, and a strong call for enhanced mental health and reconciliation programs. The cross‐sectional design limits causal inferences, but the robust sample provides critical insights into the movement's effects, guiding future research and interventions.

### 5.1 Sociodemographic Characteristics

The sample consisted of 464 students from public (87.93%) and private (12.07%) universities, aged 19–30 years (51.51% aged 19–22, 38.58% aged 23–26, and 9.91% aged 27–30). The student body comprised 57.76% men and 42.24% women, with 83.62% undergraduates and 16.38% postgraduates. Notably, 81.90% of respondents participated in the July Students’ Movement, whereas 18.10% did not, ensuring diverse perspectives on its impacts (see Table 2). This composition represents a broad cross‐section of Bangladesh's university population, which is critical for understanding the movement's effects across different demographic groups.

**TABLE 2:** Sociodemographic characteristics of survey respondents (*N* = 464).

Variable	*N*	%
**Age**		
19–22	239	51.51
23–26	179	38.58
27–30	46	9.91
**Gender**		
Men	268	57.76
Women	196	42.24
**University type**		
Government	408	87.93
Private	56	12.07
**Level of study**		
Graduate	388	83.62
Postgraduate	76	16.38
**Participate in movement**		
Yes	379	81.90
No	85	18.10

*Source:* Author's creation.

### 5.2 Psychological Impacts

Guided by trauma theory [38], the survey assessed psychological distress following the movement (see Table 3). High anxiety when recalling movement events was reported by 60.3% of students (*M* = 4.2, SD = 0.8), indicating a profound emotional toll. Sleep disturbances affected 36.2% (*M* = 3.6, SD = 1.0), whereas feelings of isolation (*M* = 3.5, SD = 1.1) and hopelessness (*M* = 3.7, SD = 0.9) were prevalent. Perceptions of campus as unsafe scored an *M* of 3.9 (SD = 0.9), with 44.8% strongly agreeing, reflecting heightened safety concerns. Headaches were also noted (*M* = 3.4, SD = 1.2), suggesting physical manifestations of stress.

**TABLE 3:** Student responses to survey items regarding mental health, coping strategies, and university support after the movement.

Item	Strongly agree	Agree	Neutral	Disagree	Strongly disagree	*M*	SD
**Academic and psychological impact**							
Difficulty focusing on academic work after the movement	54 (46.6%)	35 (30.2%)	18 (15.5%)	6 (5.2%)	3 (2.6%)	3.8	0.97
Anxiety or stress when recalling movement events	70 (60.3%)	30 (25.9%)	10 (8.6%)	4 (3.4%)	2 (1.7%)	4.2	0.87
Sleep disturbances (insomnia, nightmares)	42 (36.2%)	35 (30.2%)	24 (20.7%)	10 (8.6%)	5 (4.3%)	3.6	1.08
Feelings of isolation or loneliness during the movement	36 (31.0%)	41 (35.3%)	24 (20.7%)	10 (8.6%)	5 (4.3%)	3.5	1.09
Feelings of hopelessness during and after the movement	47 (40.5%)	35 (30.2%)	24 (20.7%)	6 (5.2%)	4 (3.4%)	3.7	1.03
Perception of the university campus as unsafe during the movement	60 (51.7%)	30 (25.9%)	18 (15.5%)	5 (4.3%)	3 (2.6%)	3.9	0.96
Headaches during and after the movement	30 (25.9%)	35 (30.2%)	30 (25.9%)	12 (10.3%)	9 (7.8%)	3.4	1.17
Anxiety caused by rumors spread on social media	64 (55.2%)	35 (30.2%)	10 (8.6%)	4 (3.4%)	3 (2.6%)	4.1	0.91
**Coping strategies**							
Used strategies like meditation, religious practices, or exercise	48 (41.4%)	41 (35.3%)	18 (15.5%)	6 (5.2%)	3 (2.6%)	3.8	0.97
Participated in counseling or mental health services	12 (10.3%)	18 (15.5%)	24 (20.7%)	36 (31.0%)	26 (22.4%)	2.5	1.22
Spent excessive time on social media and online platforms	60 (51.7%)	35 (30.2%)	12 (10.3%)	5 (4.3%)	4 (3.4%)	4.0	0.94
Engaged in activities like music, poetry, or sports to cope with anxiety	42 (36.2%)	35 (30.2%)	24 (20.7%)	10 (8.6%)	5 (4.3%)	3.7	1.08
Avoided thinking or discussing the movement to control anxiety	24 (20.7%)	30 (25.9%)	36 (31.0%)	18 (15.5%)	8 (6.9%)	3.2	1.16
**University support and reconciliation**							
Programs to rebuild relationships among students, teachers, and staffs	53 (45.7%)	36 (31.0%)	18 (15.5%)	6 (5.2%)	3 (2.6%)	3.9	0.95
Support programs for affected students	47 (40.5%)	35 (30.2%)	24 (20.7%)	6 (5.2%)	4 (3.4%)	3.7	1.02
Participation in university‐organized dialogues improved mental health	42 (36.2%)	30 (25.9%)	29 (25.0%)	9 (7.8%)	6 (5.2%)	3.5	1.12
The feeling of unity and solidarity with peers after the movement	60 (51.7%)	35 (30.2%)	12 (10.3%)	5 (4.3%)	4 (3.4%)	4.0	0.94
Belief that the university should take more initiatives for mental healing	72 (62.1%)	30 (25.9%)	10 (8.6%)	3 (2.6%)	1 (0.9%)	4.2	0.83
Inclusion of student perspectives in reconciliation efforts	47 (40.5%)	41 (35.3%)	18 (15.5%)	6 (5.2%)	4 (3.4%)	3.8	0.99

*Source:* Author's creation.

### 5.3 Coping Mechanisms

Informed by coping theory [42], students’ coping strategies were predominantly informal. Support from friends and family was the most relied‐upon mechanism, with 56.0% strongly agreeing (*M* = 4.1, SD = 0.7). Engagement in activities such as meditation, exercise, or creative pursuits was moderate (*M* = 3.8, SD = 0.9), whereas excessive social media use was reported as an everyday occurrence (51.7% strongly agreed, *M* = 4.0, SD = 0.8), often exacerbating stress. Formal counseling services were underutilized, with only 10.3% strongly agreeing to their use (*M* = 2.5, SD = 1.1), highlighting barriers such as stigma or limited access in Bangladesh (see Table 3).

### 5.4 University Initiatives and Reconciliation

Drawing on reconciliation theory [49], the survey evaluated perceptions of university‐led efforts. Programs to rebuild relationships were moderately valued (*M* = 3.9, SD = 0.8), with 45.7% strongly agreeing. Support initiatives for affected students scored an *M* of 3.7 (SD = 0.9), indicating room for improvement. Participation in university dialogues had a lower impact on mental health (*M* = 3.5, SD = 1.0), with only 30.2% strongly agreeing. However, a strong sense of unity with peers emerged (51.7% strongly agree, *M* = 4.0, SD = 0.7), and 62.1% strongly supported enhanced mental health initiatives (*M* = 4.2, SD = 0.8). Including student perspectives in reconciliation efforts yielded a mean score of 3.8 (SD = 0.9), indicating a need for more participatory approaches (see Table 3). These findings underscore the crucial role of institutional support in facilitating post‐movement recovery.

### 5.5 Correlation Analysis

Correlation analyses explored demographic influences on psychological responses (see Table 4). Older students reported lower anxiety related to social media rumors (*r* = −0.22, *p* < 0.01), indicating a reduction in anxiety with age, and fewer campus safety concerns (*r* = −0.20, *p* < 0.01). Negative correlations were also found between age and hopelessness (*r* = −0.17, *p* < 0.05) and anxiety, as well as recalling events (*r* = −0.18, *p* < 0.05), suggesting greater resilience among older students. Female students (coded as 2) exhibited higher stress levels, with positive correlations observed for anxiety stemming from social media rumors (*r* = 0.18, *p* < 0.01), campus safety concerns (*r* = 0.15, *p* < 0.05), and hopelessness (*r* = 0.13, *p* < 0.05). Postgraduate students exhibited stronger coping mechanisms (*r* = 0.16, *p* < 0.05) in terms of support from friends/family, whereas government university students reported greater access to peer support (*r* = 0.14, *p* < 0.05).

**TABLE 4:** Correlation analysis of sociodemographic factors and psychological impacts (*N* = 464).

	Age	Gender	Univ. type	Study level
**Psychological impact variable**				
Participation in Student Movement	−0.12	0.08	−0.15	0.10
Difficulty focusing on academics	0.05	−0.10	0.07	−0.08
Anxiety/Stress when recalling events	−0.18	0.12	−0.10	0.15
Sleep disturbances	−0.10	0.05	−0.08	0.12
Feelings of isolation/loneliness	−0.15	0.10	−0.12	0.14
Perception of campus as unsafe	−0.20	0.15	−0.18	0.18
Feelings of hopelessness	−0.17	0.13	−0.14	0.16
Headaches	−0.10	0.08	−0.09	0.11
Anxiety from social media rumors	−0.22	0.18	−0.20	0.20
**Coping strategies**				
Sought support from friends/family	0.10	−0.05	0.08	−0.07
Used coping strategies	0.12	−0.08	0.10	−0.09
Participated in counseling	0.15	−0.10	0.12	−0.11
Spent excessive time on social media	−0.18	0.15	−0.16	0.17
Engaged in recreational activities	0.10	−0.07	0.09	−0.08
**University support perceptions**				
University reconciliation programs	0.08	−0.05	0.07	−0.06
Support programs for affected students	0.10	−0.07	0.09	−0.08
Participation in dialogues improved	0.12	−0.08	0.10	−0.09
Feeling of unity with peers	0.15	−0.10	0.12	−0.11
Need for more initiatives	0.18	−0.12	0.15	−0.14

*Note:* gender: male = 1, female = 2; university type: government = 1, private = 2; study level: undergraduate = 1, postgraduate = 2. All psychological variables were measured on 1–5 Likert scales. Correlations are significant at *p* < 0.05.

*Source:* Author's creation.

### 5.6 Regression Analysis

Multiple regression analysis identified predictors of psychological responses (see Table 5). Gender (*β* = 0.3006, *p* = 0.028) and study level (*β* = 0.9977, *p* = 0.022) were significant predictors, indicating that female students and postgraduates experienced more substantial psychological impacts. Age was marginally significant (*β* = −0.12, *p* = 0.060), suggesting a trend toward reduced distress with age. University type and movement participation were not significant predictors (*p* > 0.05). The model's *R* ^2^ = 0.063 suggests that other unmeasured factors (e.g., prior trauma and quality of social support) may also influence responses.

**TABLE 5:** OLS regression results.

OLS regression results			
Dep. variable	Likert_Avg	*R*‐squared	0.063
Model	OLS	Adj. *R*‐squared	0.029
Method	Least squares	*F*‐statistic	1.837
Date	Monday, February 03, 2025	Prob (*F*‐statistic)	0.127
Time	21:36:49	Log‐Likelihood	−109.35
No. observations	464	AIC	228.7
Df residuals	459	BIC	242.4
Df model	5		
Covariance type	Nonrobust		

	Coef.	Std. err.	*t*	*p *> |*t*|	[0.025]	[0.975]
Age^a^	0.0659	0.035	1.901	0.060	−0.003	0.135
Gender	0.3006	0.135	2.227	0.028	0.033	0.568
University type	−0.0326	0.227	−0.144	0.886	−0.482	0.417
Study level	0.9977	0.429	2.324	0.022	0.147	1.849
Participation	−0.2456	0.171	−1.436	0.154	−0.585	0.093
Omnibus		4.835	Durbin–Watson			1.631
Prob (omnibus)		0.089	Jarque–Bera (JB)			4.353
Skew		−0.368	Prob (JB)			0.113
Kurtosis		3.612	Cond. no.			164.

*Source:* Author's creation.

^a^Standard errors assume that the covariance matrix of the errors is correctly specified.

### 5.7 Pearson Correlation Coefficients

**FIGURE 3:**
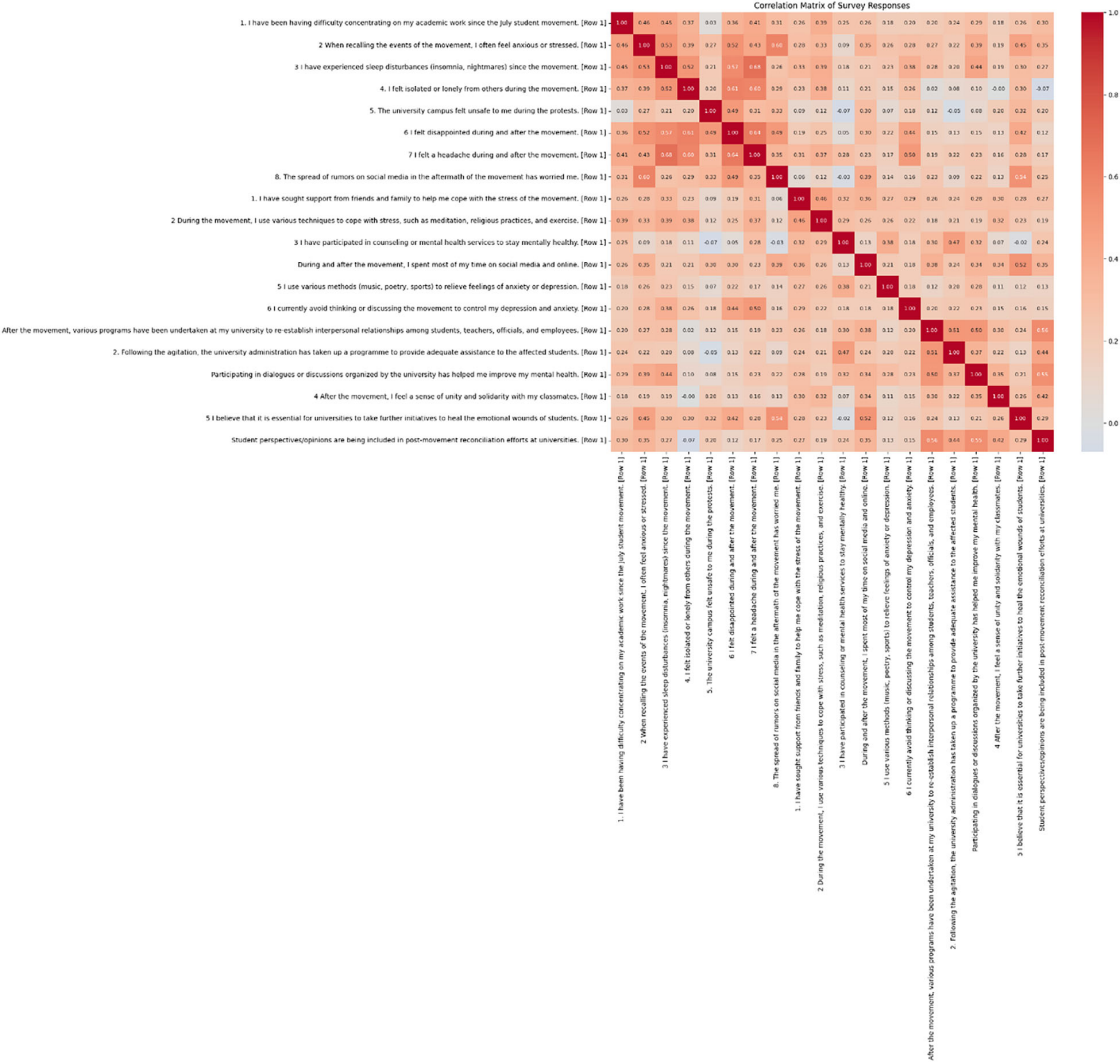
Correlations among survey questions. *Source:* Author's creation.

Strong positive correlations were found between academic difficulties and emotional stress (*r* = 0.65, *p* < 0.001) and sleep disturbances (*r* = 0.58, *p* < 0.001), reflecting their interconnected impact. Participation in supportive activities (e.g., peer dialogues) correlated moderately with improved mental health (*r* = 0.52, *p* < 0.001), and university mental health efforts showed significant associations with emotional well‐being (*r* = 0.60, *p* < 0.001). A sense of community solidarity was strongly correlated with resilience (*r* = 0.55, *p* < 0.001), as visualized in the heatmap (Figure 3).

## 6 Discussion

The findings from this study on the psychological impacts of the July Students’ Movement in Bangladesh align with and extend recent global research on the mental health consequences of youth activism while offering unique insights into the sociocultural context of Bangladesh. The high prevalence of anxiety (*M* = 4.2) and stress reported by 60.3% of participants when recalling movement events corroborates recent studies on sociopolitical activism. For instance, Wrigley et al. [26] found that climate justice advocates in Western Australia experienced significant emotional distress, including anxiety and burnout, due to their activism, a pattern echoed in our findings. Similarly, López et al. [16] highlighted the nonlinear relationship between civic engagement and mental health, noting that activism can lead to both empowerment and psychological strain, particularly in high‐stakes environments. The July Students’ Movement, marked by intense sociopolitical unrest, mirrors these dynamics, with students reporting sleep disturbances (*M* = 3.6) and feelings of hopelessness (*M* = 3.7), consistent with the emotional toll documented in movements like the Hong Kong Umbrella Movement [57] and South Africa's #FeesMustFall protests [58].

However, a key distinction in this study is the limited utilization of formal counseling services, with only 10.3% of respondents engaging in such support (*M* = 2.5). This contrasts sharply with findings from Western contexts, where mental health service uptake is higher due to greater accessibility and lower stigma. For example, a 2024 study by Lipson et al. on US college students reported a 35% increase in mental health service utilization following activism, attributed to a robust campus counseling infrastructure. In Bangladesh, cultural stigma and limited mental health resources likely contribute to this gap, aligning with Patel et al.’s [59] observations on barriers to professional mental health care in South Asia. This discrepancy underscores the need for culturally tailored interventions, such as community‐based peer support, which was highly valued by participants (*M* = 4.1 for seeking support from friends and family).

The reliance on informal support systems, including friends, family, and activities such as meditation or exercise (*M* = 3.8), aligns with findings from other contexts in the Global South. For instance, Kashif et al. [60] noted that Pakistani university students heavily depend on familial and peer networks to cope with academic and sociopolitical stress due to limited access to formal services. This contrasts with European studies, such as Drury and Reicher's [61] analysis of UK protest movements, where structured interventions like therapy groups were more prevalent. The preference for informal coping in Bangladesh underscores the relevance of coping theory [42], which emphasizes adaptive strategies such as social support. This study extends coping theory by demonstrating how community connections, particularly post‐movement solidarity (*M* = 4.0), enhance coping self‐efficacy in a non‐Western context, supporting Dost's [47] findings on the role of belongingness in student well‐being.

Social media's dual role as both a stressor and a platform for solidarity (*M* = 4.1 for anxiety from rumors, 51.7% reporting excessive use) is a critical finding that resonates with global trends. Adewunmi [35] found that social media amplified anxiety during Nigeria's #EndSARS protests by spreading misinformation, yet also fostered collective resilience through online support networks. Similarly, our study highlights how social media exacerbated anxiety through rumors (55.2% strongly agreeing) while enabling peer solidarity, reinforcing Tufekci's [62] concept of networked protest's “power and fragility.” This duality contributes to trauma theory [38] by illustrating how digital environments can act as secondary trauma sources, necessitating trauma‐informed digital literacy programs as suggested by Bar et al. [40].

The call for enhanced university‐led reconciliation initiatives (62.1% strongly agreeing, *M* = 4.2) aligns with reconciliation theory [49] and findings from post‐conflict settings. For example, Chen et al. [50] demonstrated that school climate and belongingness mediate negative emotions in post‐conflict settings, supporting our finding that inclusive reconciliation efforts (*M* = 3.8) are critical for healing. However, the relatively low *M* score for student inclusion in reconciliation (3.8) suggests a gap in participatory approaches, mirroring challenges in Sri Lanka's post‐conflict programs, where top‐down initiatives often failed to address youth needs [63]. This study advances reconciliation theory by emphasizing the need for student‐driven reconciliation processes, particularly in educational settings, to foster long‐term psychological recovery.

Gender and age differences in psychological responses further enrich the theoretical framework. Female students reported higher anxiety (*r* = 0.18 with social media rumors, *r* = 0.15 with campus safety perceptions), consistent with Yao et al.’s [51] findings on gender‐based mental health disparities among Chinese university students. Younger students (aged 19–22) exhibited greater distress, aligning with Barber's [64] research on Palestinian youth, where developmental vulnerabilities amplified activism‐related stress. These findings contribute to trauma theory by highlighting how demographic factors mediate trauma responses, supporting Bar et al.’s [40] social‐ecological perspective on trauma.

In contrast to studies like Saavedra et al. [22], which found that marginalized identities (e.g., queer Black girls) amplify the psychological costs of activism, our study suggests that older students and males in Bangladesh exhibited greater resilience, possibly due to stronger social networks or prior coping experiences. This contrast highlights the context‐specific nature of activism's mental health impacts, enriching Positive Developmental Theory [20] by showing how supportive community contexts can mitigate distress in non‐Western settings.

This research makes a significant contribution to trauma, coping, and reconciliation theories by providing empirical evidence from a South Asian context, where such studies are scarce. It extends trauma theory by identifying social media as a novel vector of trauma, supports coping theory by highlighting the efficacy of informal support systems, and advances reconciliation theory by advocating for participatory, student‐centered approaches. Unlike Western‐centric studies, it underscores the need for culturally sensitive interventions, addressing gaps in mental health infrastructure and stigma. By integrating these findings, this study provides a nuanced understanding of the psychological toll and recovery processes associated with youth activism, with implications for policymakers and educators worldwide.

### 6.1 Implications

This study highlights the pressing need for policy interventions to support the psychological well‐being of university students affected by sociopolitical movements, such as the July Students’ Movement in Bangladesh. The findings offer actionable guidance for creating supportive, resilient, and inclusive environments for student activists, addressing both immediate mental health needs and long‐term reconciliation efforts.

The low utilization of formal counseling services (10.3% of respondents, *M* = 2.5) underscores a critical gap in mental health support, likely due to cultural stigma and limited access in Bangladesh. Universities must prioritize establishing or expanding mental health centers with culturally sensitive approaches to reduce stigma and encourage help‐seeking behavior. Integrating mental health education into curricula can normalize discussions about psychological well‐being, equipping students with tools to manage stress and trauma. Employing mental health professionals, who are fluent in local languages and contexts, can enhance accessibility and relatability, thereby addressing the barriers identified in the study [59]. These interventions align with coping theory [42] by fostering adaptive strategies and building resilience through accessible support systems.

The heavy reliance on informal support networks, such as friends and family (56.0% strongly agreeing, *M* = 4.1), highlights their value but also underscores the need for complementary, structured programs. Universities should implement peer‐led counseling and community‐based support groups to bridge the gap between informal and professional care. Training students as mental health first responders can empower them to provide initial support, fostering a culture of solidarity and care, as evidenced by the strong sense of unity reported (*M* = 4.0). Such initiatives support reconciliation theory [49] by leveraging community connections to promote collective healing and reduce feelings of isolation (*M* = 3.5).

Social media's dual role as a source of stress (55.2% reporting anxiety from rumors, *M* = 4.1) and a platform for solidarity necessitates targeted interventions. Digital literacy programs can equip students to critically evaluate online content, combat misinformation, and manage digital well‐being, addressing the anxiety amplified by social media rumors. Universities could partner with social media platforms to flag harmful content and promote mental health resources, creating safe online spaces for students to share experiences and access reliable support. These measures align with trauma theory [38] by mitigating secondary trauma from digital environments, enhancing resilience as suggested by Bar et al. [40].

The strong demand for university‐led reconciliation initiatives (62.1% strongly agreeing, *M* = 4.2) underscores the need for inclusive and participatory programs. Universities should develop peacebuilding efforts, such as workshops, dialogue sessions, and storytelling forums, that center diverse student voices to rebuild trust and foster unity. The moderate score for including student perspectives (*M* = 3.8) suggests that current efforts may lack inclusivity, a finding consistent with challenges in post‐conflict settings, such as Sri Lanka [63]. Policies that adopt participatory approaches can enhance reconciliation efforts, aligning with reconciliation theory by addressing both individual and collective psychological needs following the movement.

Significant demographic disparities, notably higher anxiety among female students (*r* = 0.18 with social media rumors) and younger students (aged 19–22), call for intersectional mental health and reconciliation strategies. Tailored interventions, such as gender‐specific support groups or targeted outreach for younger students, can address these vulnerabilities. Students at private institutions, who reported less access to peer support, may benefit from enhanced community‐building initiatives. Embedding these policies within broader educational reforms can promote holistic student well‐being, supporting Positive Developmental Theory [20] by fostering supportive developmental contexts.

The July Students’ Movement emphasizes the profound psychological challenges and resilience of young activists. By implementing these evidence‐based recommendations, universities and policymakers can create environments that address mental health needs, promote reconciliation, and empower student communities to navigate future sociopolitical challenges. These efforts will not only enhance individual well‐being but also strengthen the capacity of student activists to drive sustainable social change, contributing to a more resilient and cohesive society.

## 7 Conclusion

The findings of this study reveal the profound psychological effects of sociopolitical activism on university students, with a particular focus on the context of the July Students’ Movement in Bangladesh. The research reveals elevated levels of anxiety, stress, and emotional distress among participants, many of whom reported difficulties in concentrating on academic responsibilities and experiencing sleep disturbances. Although support from friends and family played an essential role in coping strategies, there was a noticeable underutilization of formal mental health services, highlighting potential barriers in accessibility and cultural acceptance of professional counseling. Social media served as a double‐edged sword, acting as both a stressor and a platform for building solidarity, thereby complicating the mental health landscape for students. Furthermore, the study emphasizes the necessity for inclusive university‐led reconciliation efforts and robust mental health initiatives aimed at healing and fostering unity within student communities.

Despite these valuable insights, some limitations of the study should be recognized. The sample was predominantly made up of students from government universities, which may limit the generalizability of the findings to private institutions or other demographic groups. Additionally, the study's cross‐sectional design limits its ability to establish causal relationships or track changes in mental health over time. Future research should aim to overcome these limitations by employing longitudinal studies to explore the long‐term effects of activism on mental health and the lasting impact of reconciliation programs. Comparative analyses across different cultural and political contexts would also provide meaningful insights into shared challenges and context‐specific strategies for mitigating the psychological burden of youth activism. Such efforts can contribute to the development of more comprehensive, global frameworks that support the well‐being of activists, taking into account their unique challenges and needs.

## Author Contributions

**Taha Husain**: study conception and design, data collection, analysis and interpretation of results, and manuscript preparation.

## Conflicts of Interest

The author declares no conflicts of interest.

## Data Availability

The dataset supporting the findings of this study is available from the corresponding author upon reasonable request.
